# High-throughput screening of dual-target inhibitors for SARS-CoV-2 main protease and papain-like protease from Chebulae Fructus: *in silico* prediction and experimental verification

**DOI:** 10.3389/fmicb.2024.1510665

**Published:** 2024-12-18

**Authors:** Changjian Wang, Yipeng Cao, Qi Yang, Xinyue Wang, Zhiying Yang, Jingjing Yang, Xinru Li, Bin Li, Yuefei Wang, Min Zhang

**Affiliations:** ^1^State Key Laboratory of Chinese Medicine Modernization, Tianjin University of Traditional Chinese Medicine, Tianjin, China; ^2^Key Laboratory of Cancer Prevention and Therapy, Tianjin’s Clinical Research Center for Cancer, National Clinical Research Center for Cancer, Tianjin Medical University Cancer Institute and Hospital, Tianjin, China; ^3^Guangzhou Laboratory, Guangzhou, China; ^4^Department of Pharmaceutical Engineering and Pharmaceutical Chemistry, College of Chemical Engineering, Qingdao University of Science and Technology, Qingdao, China; ^5^Haihe Laboratory of Modern Chinese Medicine, Tianjin, China

**Keywords:** SARS-CoV-2, main protease, papain-like protease, 1,2,3,4,6-penta-*O*-galloyl-*β*-D-glucose (PGG), 1,2,3,6-tetra-*O*-galloyl-β-D-glucose (TGG)

## Abstract

**Background:**

The unavoidable propagation of the coronavirus disease 2019 (COVID-19), caused by the severe acute respiratory syndrome coronavirus 2 (SARS-CoV-2), has underscored the urgent requirement for efficacious therapeutic agents. The dried fruit of *Terminalia chebula* Retz., namely Chebulae Fructus, is widely used for treating bacterial and viral infectious diseases, which was witnessed to perform anti-SARS-CoV-2 activity in recommended Chinese patent medicine.

**Aim:**

SARS-CoV-2 main protease (M^pro^) and papain-like protease (PL^pro^) present essential effects on SARS-CoV-2 replication and transcription, considering as the attractive targets for therapeutic intervention. In this study, we focused on the dual-target to obtain broad-spectrum antiviral candidates from Chebulae Fructus.

**Methods:**

The identified compounds from Chebulae Fructus were used to build a library in a previous study, which were evaluated by molecular docking to screen potential antiviral agents. The SARS-CoV-2 M^pro^ and PL^pro^ were expressed in *E. coli* cells and purified. Fluorescence resonance energy transfer (FRET) and surface plasmon resonance (SPR) were utilized to verify the affinity with dual targets. SARS-CoV-2 wild-type, Omicron BA.5 and Omicron EG.5 variants were employed to validate their antiviral activities *in vitro*. Molecular dynamics simulation was conducted via Gromacs 2022 software in 500 ns to unveil the conformation stability.

**Results:**

Targeting on M^pro^ and PL^pro^, eight compounds were screened as the potential dual-target inhibitors in molecular docking. In FRET and SPR assays, 1,2,3,4,6-penta-*O*-galloyl-*β*-D-glucose (PGG) and 1,2,3,6-tetra-*O*-galloyl-*β*-D-glucose (TGG) showed good inhibitory activities with IC_50_ values ranging from 1.33 to 27.37 μM, and affinity with *K_D_* values ranging from 0.442 to 0.776 μM. Satisfactorily, both PGG and TGG display antiviral activity *in vitro* with EC_50_ values ranging from 3.20 to 37.29 μM, suggesting as the promising candidates against SARS-CoV-2. In molecular dynamics simulation study, the complexes of M^pro^-PGG, M^pro^-TGG, PL^pro^-PGG, and PL^pro^-TGG exhibited stability over 500 ns period, unveiling the potential interactions.

**Conclusion:**

PGG and TGG are the promising dual-target inhibitors of SARS-CoV-2, which may avoid drug resistance and have a good development prospect. The outcomes of this study provide an effective strategy to systematically explore the antiviral bioactive compounds from Chebulae Fructus.

## 1 Introduction

The quest for curbing the global proliferation of coronavirus disease 2019 (COVID-19), triggered by the severe acute respiratory syndrome coronavirus 2 (SARS-CoV-2), still represents a critical challenge. Since its initial outbreak in late 2019, it has led to over 775 million confirmed cases and 7.05 million deaths worldwide as of August 2024 (WHO, n.d.). The advent of vaccines was a pivotal milestone in preventing the spread of COVID-19. The emergence of SARS-CoV-2 variants has been accompanied by a notable immune escape, while the protective effects of vaccines have been found to be relatively weak (Jackson et al., 2020; Li et al., 2022; Yu et al., 2020). Moreover, the pace of antiviral drug development has not kept up with the rapid mutation rate of SARS-CoV-2 (Beigel et al., 2020). It underscores the importance of identifying more effective and biosafe broad-spectrum antiviral agents to treat COVID-19 effectively.

In the battlefield against SARS-CoV-2, the main protease (M^pro^) (Liu et al., 2022) and the papain-like protease (PL^pro^) (Zang et al., 2023) devote essential part to cleaving the polyproteins pp1a and pp1ab to 16 non-structural proteins (Nsps), which is crucial for SARS-CoV-2 replication. M^pro^ comprises three domains (domains I, II, and III) and takes charge of 11 cleavage sites at least (Jin et al., 2020). Given high conservation in the active site across coronaviridae viruses and non-homology to mammalian proteins, M^pro^ is regarded as the most crucial target for the development of broad-spectrum antiviral candidates. In a previous study, the active unit of M^pro^ is a homodimer, whose substrate-binding site located within the C145-H41 catalytic dyad between domain I and II (Jin et al., 2020). The catalytic pocket is an important region for inhibitor binding. PL^pro^ is another cleavage protease, belonging to a cysteine protease, and takes charge of other three cleavage sites to release Nsp1, Nsp2 and Nsp3. The PL^pro^ contains two potential inhibitor binding pockets, including a classical catalytic triad (C111-H272-A286) and the BL2 domain. Unlike M^pro^, the PL^pro^ catalytic domain is normally off but opens when substrates close to it, making it harder for molecules to bind and giving low inhibitory activity. However, PL^pro^ is capable of removing signaling proteins and ubiquitin-like interferon-stimulated gene 15 (ISG15) in the host, thereby suppressing innate immune responses (Shin et al., 2020). It gives the PL^pro^ inhibitors with both antiviral and anti-inflammatory activity (Osipiuk et al., 2021), which is an important feature differing from M^pro^ inhibitors. Therefore, dual-target inhibitors of M^pro^ and PL^pro^ offer a promising therapeutic strategy, akin to a combination therapy within a single medication, potentially circumventing drug resistance more effectively than single-target drugs in the fight against viral infections (Yang et al., 2005). Recent research has shown that simultaneous inhibition of M^pro^ and PL^pro^ is markedly more efficacious in combating SARS-CoV-2 and its variants (Narayanan et al., 2022). Therefore, we focused on SARS-CoV-2 M^pro^ and PL^pro^ to develop dual-target inhibitors. However, despite both enzymes being cysteine proteases, their substrate specificities and active site configurations differ, posing challenges for screening effective dual-target inhibitors (Rut et al., 2020).

Chinese herbal medicine is a great treasure, supplying numerous strategies to conquer diseases. In COVID-19 treatment, due to the wide application of traditional Chinese medicine, the mortality and severe disease incidence reduce significantly (Lyu et al., 2023). The dried fruit of *Terminalia chebula* Retz., namely Chebulae Fructus (*CF*), is referred to as the king of Tibetan medicine (Nigam et al., 2020), and is widely used in many countries for its antibacterial and antiviral effects, such as India, Bhutan, Maldives, Nepal and China (Bidikar et al., 2022). Notably, *CF* extract displays broad-spectrum antiviral activities, including herpes simplex virus-2 (Kesharwani et al., 2017), influenza A virus strains (Li et al., 2020), human immunodeficiency virus (Ahn et al., 2002), and SARS-CoV-2 (Upadhyay et al., 2020). Several effective Chinese patent medicines with *CF* as the main component were, respectively, recommended by the National Medical Products Administration (NMPA) and the Joint Prevention and Control Mechanism of the State Council, such as Tibetan medicine influenza pills, Cuitang granules, and Qingyan Dropping pills. With natural products’ diverse structures and complex compositions, they are invaluable for antiviral drug discovery. It is imperative to rapidly identify bioactive compounds from *CF* and clarify their antiviral mechanism, supplying the structural guidance for lead compound design, which is crucial for the development of antiviral drugs.

In this study, a total of 122 compounds derived from *CF* were subjected to molecular docking to assess their binding energy with both M^pro^ and PL^pro^. Of these, eight compounds exhibited strong binding affinity. Specifically, 1,2,3,4,6-penta-*O*-galloyl-*β*-D-glucose (PGG) and 1,2,3,6-tetra-*O*-galloyl-*β*-D-glucose (TGG) demonstrated high enzymatic activity against M^pro^ and PL^pro^ by fluorescence resonance energy transfer (FRET). Furthermore, through surface plasmon resonance (SPR) assay, PGG and TGG possess good binding affinities for M^pro^ and PL^pro^. They exhibited potent antiviral activity against both SARS-CoV-2 wild-type strain, Omicron BA.5 and Omicron EG.5 variant in Vero E6 cells. In the end, molecular dynamics simulation was employed to unveil the potential binding mechanism, suggesting that PGG and TGG could serve as promising dual-target inhibitors of M^pro^ and PL^pro^ against SARS-CoV-2.

## 2 Materials and methods

### 2.1 Chemicals and reagents

The tested compounds (PGG, TGG, punicalin, and 1,3,6-tri-*O*-galloylglucose) and three positive controls (PF-07321332, GRL0617, and S-217622) were acquired from Shanghai Yuanye Bio-Technology Co., Ltd. (Shanghai, China) with purity of 98% by HPLC. CM5 sensor chips, amine-coupling kit, and running buffer were purchased from General Electric Company (Boston, MA, USA). Dulbecco’s modified Eagle’s medium and fetal bovine serum were purchased from Gibco Invitrogen Corp. (New York, USA).

### 2.2 Molecular docking

The crystal structures of SARS-CoV-2 M^pro^ (PDB ID: 6 LU7), SARS-CoV-2 PL^pro^ (PDB ID: 7CJM), MERS-CoV M^pro^ (PDB ID: 9BOO), and HCoV 229E M^pro^ (PDB ID: 7YRZ) were obtained from the RCSB PDB.^1^ The 122 compounds in *CF*, which were identified in a previous study (Wang et al., 2023), were employed as ligands. The structural details of the ligands were obtained from PubChem^2^ database and ChemDraw in SDF or mol2 formats, respectively.

Ligands and proteins were prepared by utilizing Discovery Studio 2020 software. The protein preparation contains the removal of extraneous water molecules, the addition of hydrogen atoms, and the repair of missing residues. The binding site was identified based on the location of the native ligand, which was subsequently excised from the protein model. Subsequently, docking simulations were conducted using the cdocker mode. The docking results were visualized by PyMOL version 2.6. N3, the native ligand of M^pro^, and GRL0617, the native ligand of PL^pro^, served as positive controls (Fu et al., 2021; Jin et al., 2020). Each ligand was docked into its respective protein.

### 2.3 Cloning, protein expression, and purification of SARS-CoV-2 M^pro^ and PL^pro^

The plasmids encoding the full-length genes of SARS-CoV-2 M^pro^ and PL^pro^ were successfully constructed and then individually transformed into *Escherichia coli* BL21 (DE3) cells. A 100 μL aliquot of competent cells was thawed on ice and mixed with either the M^pro^ or PL^pro^ plasmid solution. The mixture was incubated on ice for 30 min before undergoing a heat shock at 42°C for 90 s. After the heat shock, 900 μL of fresh LB medium was added to the cells to facilitate recovery at 37°C 220 rpm for 1 h. The supernatant was then cleanly and quickly pipetted off after centrifugation. The recovered cultures were spread onto LB agar plates containing the appropriate antibiotic at 37°C for 12–16 h. As s shown in Supplementary Figure S1, these transformed cells were then cultured in Luria broth medium supplemented with 100 μg/mL ampicillin for M^pro^ expression and 50 μg/mL kanamycin for PL^pro^ expression. Cultivation was carried out at 37°C for 6–8 h. Subsequently, 500 μM isopropyl-beta-D-thiogalactopyranoside (IPTG) was added to induce protein expression at 16°C for 16–20 h. Following induction, the cells were harvested by centrifugation at 1,500 × *g* for 20 min at 4°C. The resultant cell pellet was lysed using a high-pressure homogenizer and centrifugation at 16,000 × *g* for 1.5 h at 4°C, and the cell supernatant was collected. The proteins were eluted by running buffer containing 20 mM Tris–HCl (pH 8.0), 150 mM NaCl, 5% glycerol, and 300 mM imidazole through the Ni-NTA affinity chromatography column (5 mL, QIAGEN, Shenzhen, China) at a flow rate of 5 mL/min. The C-terminal 6 × His tag of M^pro^ was excised by human rhinovirus 3C protease, while the small ubiquitin-like modifier moiety attached to PL^pro^ was cleaved with small ubiquitin-like modifier enzyme. For the ion exchange chromatography, a HiTrap™ Q HP column (5 mL, GE Healthcare, Little Chalfont, UK) at a flow rate of 1 mL/min was utilized with a buffer system comprising 20 mM Tris–HCl (pH 8.0) and 1 M NaCl. The elution was performed using a gradient of NaCl concentration from 50 mM to 1 M to effectively separate the proteins based on their different charges. The size-exclusion chromatography was conducted on a Superdex 200 Increase 10/300 GL column (GE Healthcare, Little Chalfont, Buckinghamshire, UK) at a flow rate of 0.5 mL/min with a buffer system containing 20 mM Tris–HCl and 150 mM NaCl at pH 8.0. Using SDS-PAGE, the purity of M^pro^ was determined to be approximately 88.3%, and PL^pro^ was about 88.5%.

### 2.4 Determination of enzymatic inhibition activities on SARS-CoV-2 M^pro^ and PL^pro^

Via a continuous kinetic assay, the enzymatic activity of SARS-CoV-2 M^pro^ was quantified by employing the specific substrate MCA-AVLQSGFR-Lys (Dnp)-Lys-NH_2_, which is synthesized by GL Biochem Co., Ltd. (Shanghai, China). This excitation and emission wavelengths were 340 nm and 460 nm, respectively. The enzymatic reaction system of M^pro^ includes 20 mM Tris–HCl, 150 mM NaCl, 2 mM DTT, 0.2 μM M^pro^ and variable substrate concentration ranging from 4 to 36 μM at a pH of 8.0. For SARS-CoV-2 PL^pro^, the substrate Dabcyl-FTLKGGYAPTKVTE-Edans was also provided by GL Biochem Co., Ltd. The excitation and emission wavelengths were 336 nm and 490 nm, respectively. The enzymatic reaction system of PL^pro^ contains 20 mM Tris–HCl, 150 mM NaCl, 10 mM DTT, 1.0 μM PL^pro^, and variable substrate concentrations ranging from 10 to 100 μM at a pH of 8.0. Fluorescence intensity was meticulously monitored using a Spark multimode microplate reader from Tecan (Männedorf, ZH, Switzerland).

For enzymatic inhibitor screening, the sample solutions of the tested compounds (40 μM) were preliminarily used to evaluate their inhibitory activities, and the compounds with inhibitory activity over 60% were employed for determining IC_50_ values (Yi et al., 2022). The enzymatic system comprised 0.2 μM M^pro^ or 1.0 μM PL^pro^, 20 μM substrate for M^pro^ or 10 μM substrate for PL^pro^, and various concentrations of the tested compounds. All experimental results were rigorously analyzed by employing GraphPad Prism 8, with triple replicates of each experiment to ensure data reliability.

### 2.5 SPR assay

The binding affinity was assessed via SPR assay, conducted on Biacore T200. M^pro^ and PL^pro^ were, respectively, immobilized onto the surface of an activated CM5 sensor chip through an amine coupling reaction. Initially, the compounds were prepared in DMSO at a concentration of 2.5 mM, serving as the standard stock solution, which was subsequently diluted 20-fold with 1.05 × PBS-P to achieve an initial concentration of 125 μM. Then, they were stepwise diluted by running buffer (1.05 × PBS-P containing 5% DMSO), yielding a range of concentrations from 0.030 μM to 125 μM. The assay conditions were optimized with a flow rate of 30 μL/min, while the association and dissociation phases were set at 120 s and 180 s, respectively. Data analysis was carried out via BIAevaluation software version 2.0 in the kinetics analysis mode to elucidate the binding properties.

### 2.6 Antiviral activity assay

The antiviral activity of the tested compounds was assessed through the inhibition of cytopathic effect (CPE) in Vero E6 cells, which was conducted in the Biosafety Level 3 (BSL-3) laboratory in Guangzhou Customs Inspection and Quarantine Technology Center. Vero E6 cells were inoculated in a 96-well plate at a density of 1 × 10^5^ cells *per* well and maintained in DMEM culture solution containing 10% fetal bovine serum, 100 μg/mL streptomycin, and 100 U/mL penicillin at 37°C with an atmosphere comprising 5% CO_2_ and 95% air. Firstly, the cytotoxicity was evaluated by the CCK8 assay to obtain the non-cytotoxicity dose, which was calculated according to the half-cytotoxic concentration (CC_50_) value. Subsequently, at the safe dose, cells were mixed with SARS-CoV-2 wild-type, Omicron BA.5 or Omicron EG.5 strain (MOI = 0.01) and the tested compounds at varying concentrations (0.0031 to 50.00 μM) and incubated for 48 h. The scores quantifying CPE were obtained by the Celigo Image Cytometer. The 50% tissue culture infectious dose (TCID_50_) was calculated by employing the Reed-Muench formula, serving as a reference to facilitate the determination of the half-maximal effective concentration (EC_50_) of the tested compounds.

### 2.7 Molecular dynamics simulation

After the evaluation of the molecular docking and experimental verification results, a molecular dynamics simulation was performed to analyze the stability of protein-ligand complexes and unveil the interaction mechanism. The CHARMM-GUI was used to construct four simulation systems, including SARS-CoV-2 M^pro^-PGG, SARS-CoV-2 M^pro^-TGG, SARS-CoV-2 PL^pro^-PGG, and SARS-CoV-2 PL^pro^-TGG. The dimensions of the simulation boxes were 12 × 12 × 12 nm^3^, with the total number of atoms varying from approximately 100,000 to 150,000. CHARMM36m force field was applied, and the simulation parameters were the same as in a previous study (Cao et al., 2023; Huang et al., 2017). A physiological environment was mimicked by incorporating 0.15 M NaCl into the simulation systems. During the simulation phase, initial energy minimization was performed using the steepest descent algorithm for 20,000 steps, then followed by a 300 ns pre-equilibration six times to optimize the system. NVT (constant volume and pressure) and NPT (constant pressure and temperature) simulations were employed by 100 ns. Finally, molecular dynamics simulation was performed for each complex at 500 ns. Electrostatic interactions were described via the Particle Mesh Ewald (PME) method, with a cutoff radius of 1.2–1.4 nm. The LINear Constraint Solver (LINCS) algorithm was employed to constrain chemical bonds. Pressure was maintained at 1 bar in the *x, y*, and *z* directions using the C-rescale pressure coupling, and temperature was kept constantly at the 310 K using the V-rescale thermostat. All simulations were conducted using the GROMACS2022 software package (Valdés-Tresanco et al., 2021). The root means square deviation (RMSD), root mean square fluctuation (RMSF), and the radius of gyration (Rg) were analyzed.

## 3 Results

### 3.1 High-throughput screening of compounds with potential inhibitory activity against M^pro^ and PL^pro^ by molecular docking

**Figure 1:**
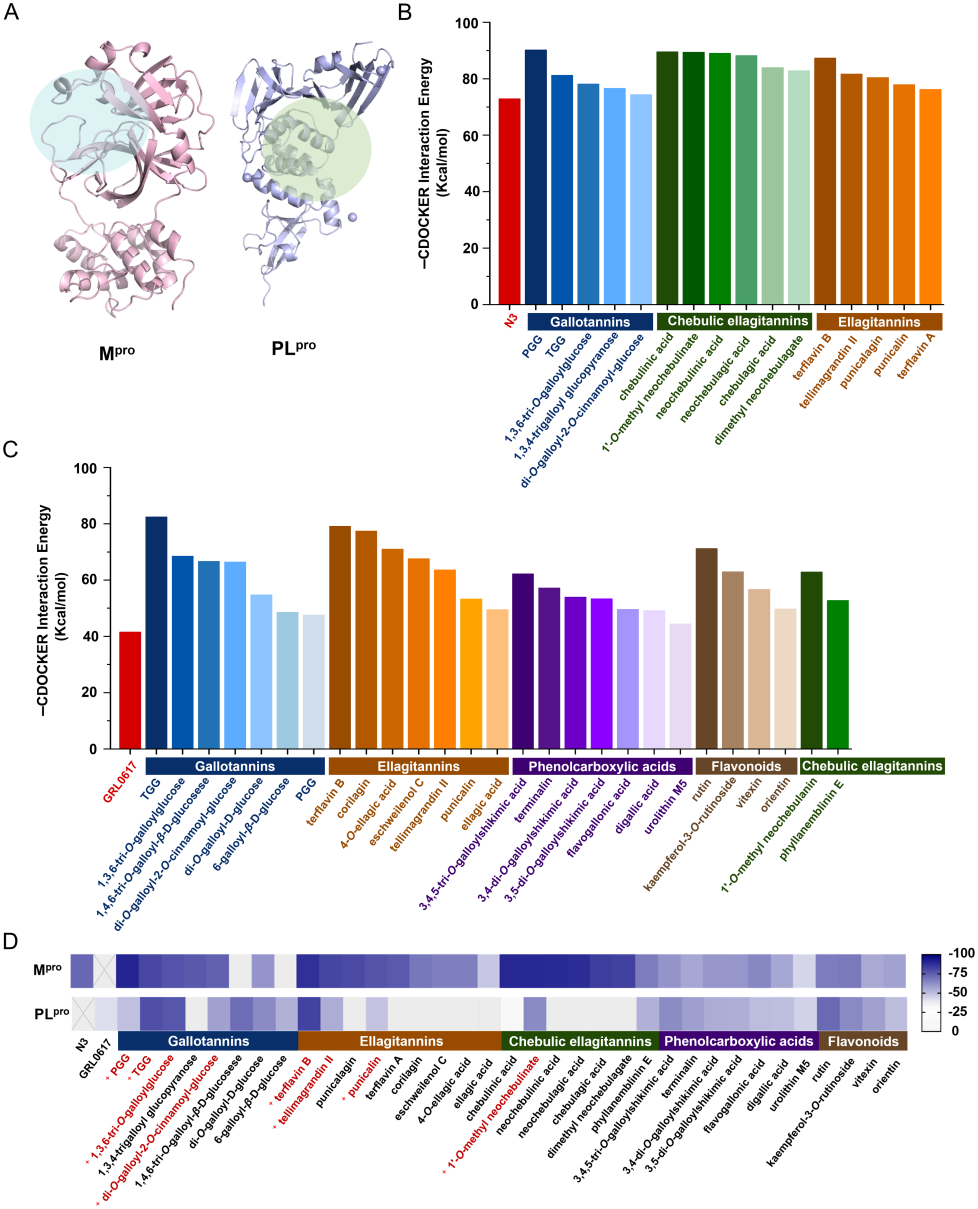
Binding affinity of the tested compounds with SARS-CoV-2 M^pro^ or PL^pro^ by molecular docking. **(A)** M^pro^ and PL^pro^ and their active pockets. The cdocker interaction energy of the tested compounds bound to M^pro^ **(B)** or PL^pro^ **(C)**. **(D)** The heatmap analysis of cdocker interaction energy to screen the potential dual-target inhibitors.

The binding energies between the tested compounds and M^pro^ or PL^pro^ were determined by Discovery Studio 2020 based on SARS-CoV-2 M^pro^ (PDB ID: 6 LU7) and PL^pro^ (PDB ID: 7CJM) crystal structures (Figure 1A), respectively. The molecular docking results showed that 16 out of the 122 compounds exhibited the lower binding affinity to M^pro^ than the positive control (N3), which were classified into three groups based on their chemical structures: gallotannins, chebulic ellagitannins, and ellagitannins (Figure 1B). Meanwhile, 27 out of the 122 compounds performed a lower binding affinity to PL^pro^ than the positive control (GRL0617), which were classified into five categories: gallotannins, ellagitannins, phenolcarboxylic acids, flavonoids, and chebulic ellagitannins (Figure 1C). To our surprise, eight compounds showed the potential dual-target inhibitory activities (Figure 1D), which need to be verified experimentally.

### 3.2 Evaluation of binding affinity with SARS-CoV-2 M^pro^ and PL^pro^ by FRET and SPR assays

Recombinant SARS-CoV-2 M^pro^ and PL^pro^ expressed in *E. coli* cells were purified (Supplementary Figures S1A,B,D,E). The enzymatic catalytic efficiency (*kcat/Km*) for substrates of SARS-CoV-2 M^pro^ and PL^pro^ was 12,516 M^−1^·S^−1^ and 1,845 M^−1^·S^−1^, respectively (Supplementary Figures 1C,F).

**Figure 2:**
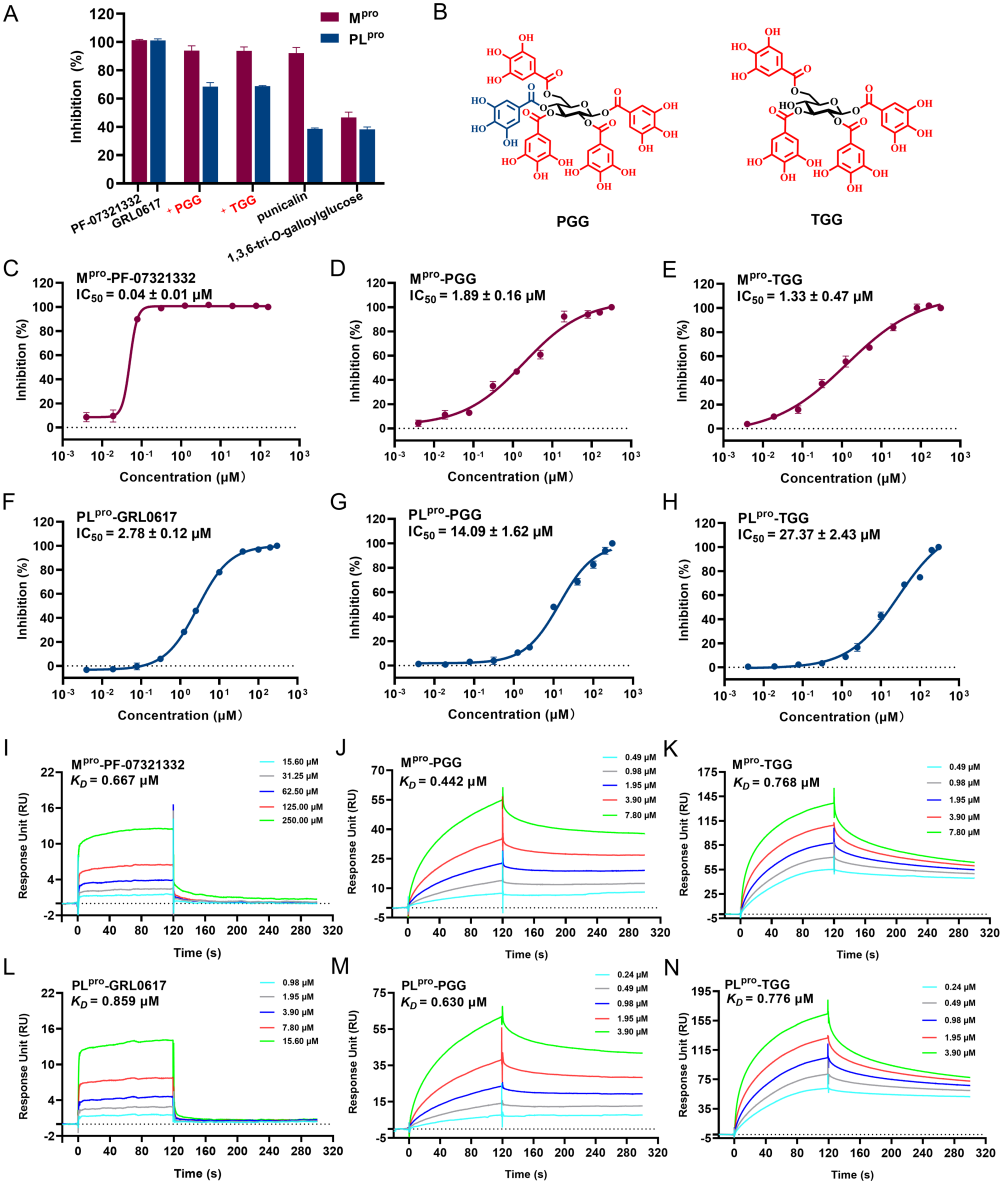
The inhibitory activities of the tested compounds against SARS-CoV-2 M^pro^ and PL^pro^. **(A)** The inhibitory activities of the tested compounds (40 μM) against M^pro^ and PL^pro^. **(B)** Chemical structures of 1,2,3,4,6-penta-*O*-galloyl-*β*-D-glucose (PGG) and 1,2,3,6-tetra-*O*-galloyl-*β*-D-glucose (TGG). The IC_50_ curves of PF-07321332 **(C)**, PGG **(D)**, and TGG **(E)** against M^pro^. The IC_50_ curves of GRL0617 **(F)**, PGG **(G)**, and TGG **(H)** against PL^pro^. SPR assay for the binding affinity of the tested PF-07321332 **(I)**, PGG **(J)**, and TGG **(K)** with M^pro^. SPR assay for binding affinity of the tested GRL0617 **(L)**, PGG **(M)**, and TGG **(N)** with PL^pro^. (mean ± SD, *n* = 3).

In the FRET assay, there are four compounds (PGG, TGG, 1,3,6-tri-*O*-galloylglucose, and punicalagin) available, which can be employed to determine the inhibitory activities. In 40 μM, PGG and TGG exhibited inhibition effects over 60% on both M^pro^ and PL^pro^ (Figure 2A). PF-07321332 is reported as the inhibitor of M^pro^, which can be employed as the positive control (Owen et al., 2021). GRL0617, used as the positive control, effectively inhibited PL^pro^ activity (Shin et al., 2020). Interestingly, these two compounds shared the similar structures. Compared with TGG, PGG possesses an additional galloyl unit at the 4th substitution base (Figure 2B). The results showed that PF-07321332, PGG, and TGG inhibited M^pro^ with IC_50_ values of 0.04 μM, 1.89 μM, and 1.33 μM, respectively (Figures 2C–E). GRL0617, PGG, and TGG inhibited PL^pro^ with IC_50_ values of 2.78 μM, 14.09 μM, and 27.37 μM, respectively (Figures 2F–H).

The binding affinities of various compounds to the M^pro^ or PL^pro^ were quantitatively assessed via SPR, a pivotal method for monitoring ligand-enzyme interactions in real time. The lower dissociation constant (*K_D_*) signifies a stronger affinity of compounds binding to the target protein, highlighting its promise as a therapeutic agent. In this study, the *K_D_* values for PF-07321332, PGG, and TGG with M^pro^ were determined to be 0.667 μM, 0.442 μM, and 0.768 μM, respectively (Figures 2I–K). The *K_D_* values for GRL0617, PGG, and TGG with PL^pro^ were evaluated to be 0.859 μM, 0.630 μM, and 0.776 μM, respectively (Figures 2L–N). In summary, the integration of FRET and SPR underscores the potential activities of PGG and TGG against SARS-CoV-2, which may hold promise in the development of novel therapeutic strategies.

### 3.3 Evaluation of antiviral activity on SARS-CoV-2 wild type, omicron BA.5 and omicron EG.5 *in vitro*

**Figure 3:**
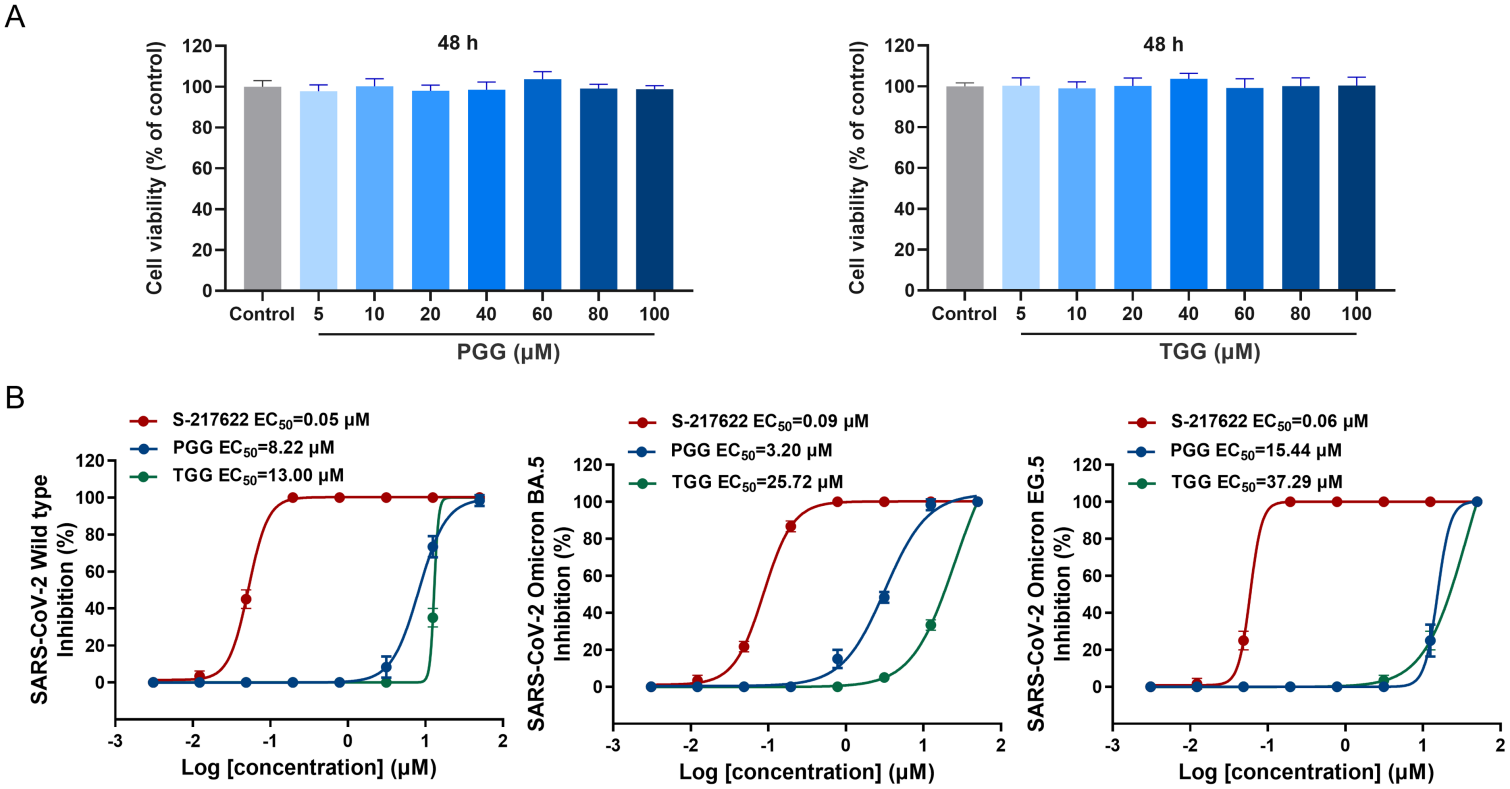
Cytotoxic effect and antiviral activity of the tested compounds. **(A)** Effect of 1,2,3,4,6-penta-*O*-galloyl-*β*-D-glucose (PGG) and 1,2,3,6-tetra-*O*-galloyl-*β*-D-glucose (TGG) on the viability of Vero E6 cells by CCK8. **(B)** The antiviral activities of S-217622, PGG, and TGG against SARS-CoV-2 wild-type, Omicron BA.5 and Omicron EG.5 *in vitro*. (mean ± SD, *n* = 3).

As the potential dual-target inhibitors, PGG and TGG showed excellent safety on Vero E6 cells with CC_50_ > 100 μM (Figure 3A). In antiviral activity assay, S-217622, a nonpeptidic inhibitor targeting M^pro^, was used as a positive control drug (Unoh et al., 2022). As illustrated in Figure 3B, the EC_50_ values for S-217622, PGG, and TGG against the SARS-CoV-2 wild-type strain are presented as 0.05 μM, 8.22 μM, and 13.00 μM, respectively. Against the Omicron BA.5 variant, these compounds display EC_50_ values of 0.09 μM for S-217622, 3.20 μM for PGG, and 25.72 μM for TGG. Furthermore, when the compounds were assessed against the Omicron EG.5 variant, the EC_50_ values are 0.06 μM for S-217622, 15.44 μM for PGG, and 37.29 μM for TGG. It suggested that PGG and TGG performed cellular protection by antiviral activity.

Also, it is hypothesized that PGG and TGG played antiviral activity mainly through inhibiting the activities of M^pro^ and PL^pro^ to prevent the viral replication. Moreover, PGG and TGG exhibit favorable solubility and stability, rendering them as promising candidates for the development of antiviral inhibitors.

### 3.4 Unveiling of the antiviral mechanism for PGG and TGG by *in silico* approach

**Figure 4:**
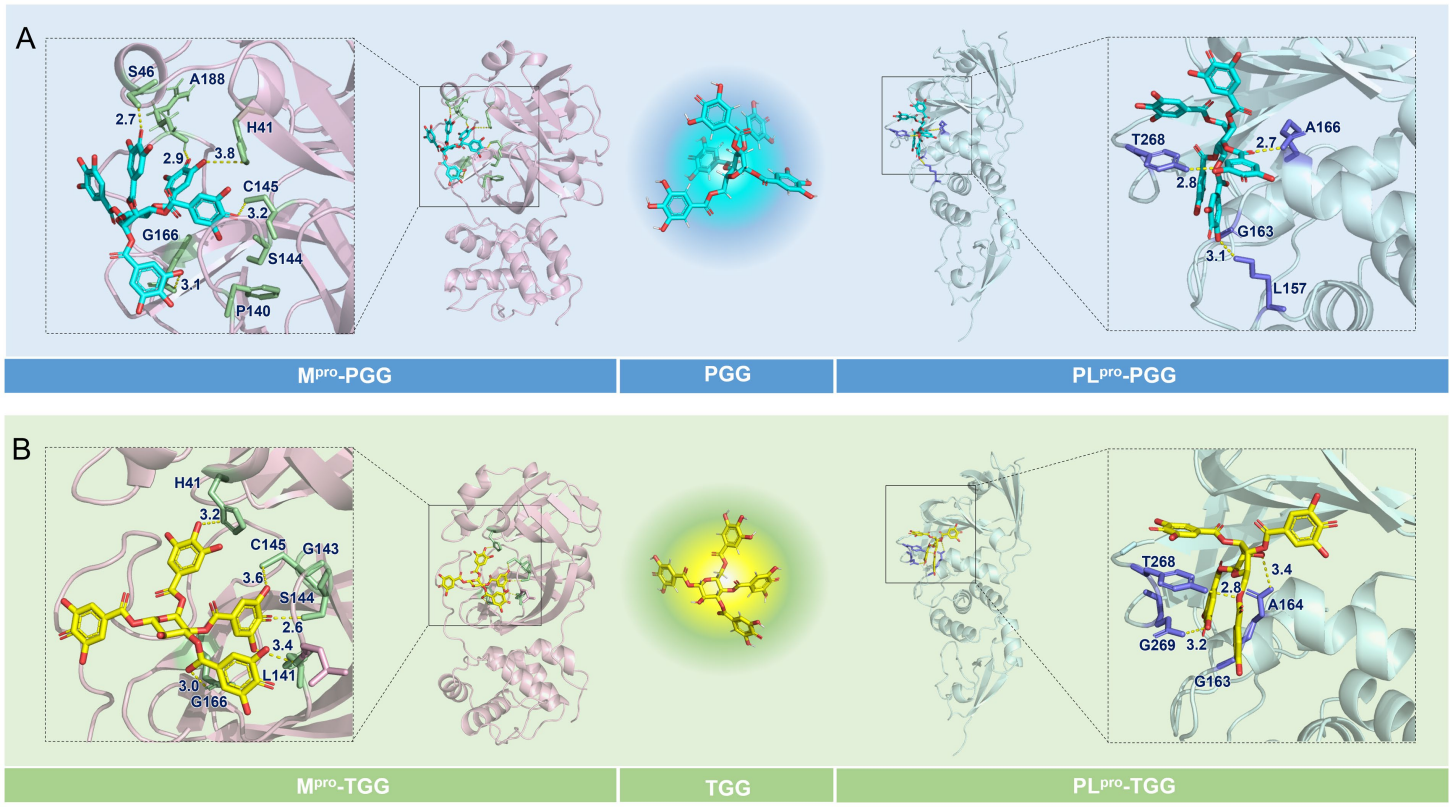
Binding modes of the focused compounds and the interesting targets. **(A)** The predicted binding modes of the M^pro^-1,2,3,4,6-penta-*O*-galloyl-*β*-D-glucose (PGG) and PL^pro^-PGG complexes. **(B)** The predicted binding modes of the M^pro^-1,2,3,6-tetra-*O*-galloyl-*β*-D-glucose (TGG) and PL^pro^-TGG complexes.

To elucidate the binding modes involved in the interaction of the focused compounds and targets, molecular docking was conducted. For SARS-CoV-2 M^pro^, the active site is defined at the C145-H41 catalytic dyad, which is originally occupied by N3. As depicted in Figures 4A,B, PGG and TGG both align in the binding pocket in preferred conformation, and the cdocker interaction energy is −90.21 and −81.24 kcal/mol, which is originated by the intermolecular hydrogen bonds. These bonds were formed with the residues H41^3.8^, S46^2.7^, C145^3.2^, G166^3.1^, and A188^2.9^ in the SARS-CoV-2 M^pro^-PGG. Similarly, in the SARS-CoV-2 M^pro^-TGG complex, hydrogen bonds were formed with the residues H41^3.2^, L141^3.4^, S144^2.6^, C145^3.6^, and G166^3.0^.

The structure of SARS-CoV-2 PL^pro^ is split into four sub-domains: the N-terminal ubiquitin-like domain (Ubl, *β*1-3), the *α*-helical thumb domain (*α*2-7), the *β*-stranded finger domain (*β*4-7), and the palm domain (*β*8-13). As shown in Figures 4A,B, PGG and TGG fit into the cleft formed between the BL2 loop and the *α*3-*α*4 loop, which is almost identical to GRL0617 (Gao et al., 2021), and the cdocker interaction energy is −49.56 and −79.11 kcal/mol, respectively. Hydrogen bonds are also the dominant interactions. In the SARS-CoV-2 PL^pro^-PGG complex, PGG formed multiple hydrogen bonds with the residues L157^3.1^, A166^2.7^, and T268^2.8^. Similarly, in the SARS-CoV-2 PL^pro^-TGG complex, TGG interacted with the residues A164^3.4^, T268^2.8^, and T269^3.2^ to form hydrogen bonds, contributing to execution of inhibition activities.

Additionally, as shown in Supplementary Figures S3A,B, docking studies of PGG and TGG with MERS-CoV M^pro^ (PDB ID: 9BOO) yielded interaction energies of −83.38 and −93.61 kcal/mol, respectively. Key interactions involve hydrogen bonds with residues S147, C148, G167, G169, and H194 for PGG, and P143, G146, G169, H175, L191, and G192 for TGG. Docking results for the HCoV-229E M^pro^ (PDB ID: 7YRZ) showed interaction energies of −83.09 and −94.67 kcal/mol for PGG and TGG, respectively, with critical hydrogen bonds involving residues H41, G142, A143, C144, G163, A186, and G191 for PGG, and A141, G142, A143, C144, G165, and G191 for TGG (Supplementary Figures 3C,D). Hence, the findings suggest that PGG and TGG may perform the potential inhibitory effects on MERS-CoV and HCoV-229E.

### 3.5 Molecular dynamics simulation

**Figure 5:**
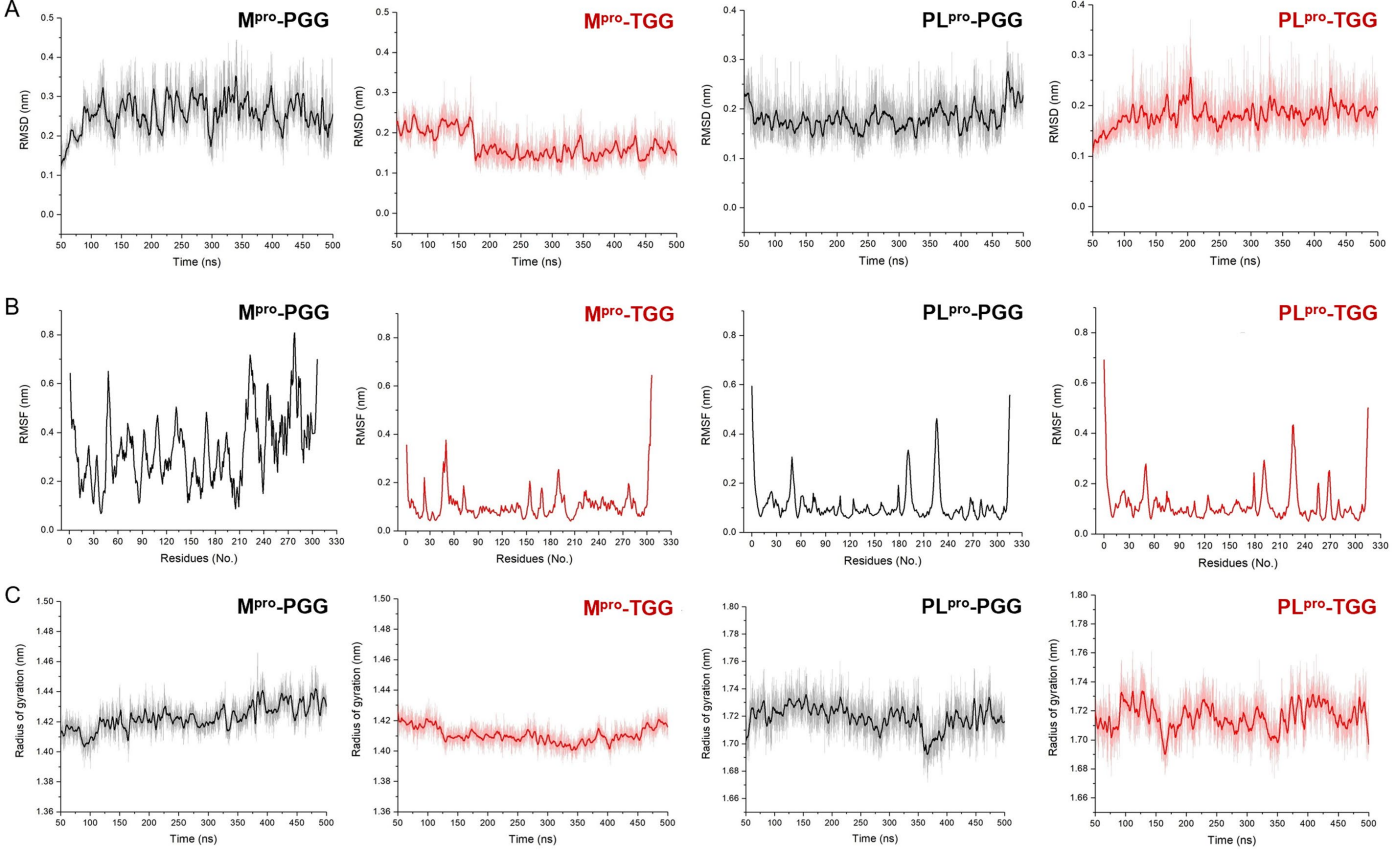
The molecular dynamics simulation for 1,2,3,4,6-penta-*O*-galloyl-*β*-D-glucose (PGG) and 1,2,3,6-tetra-*O*-galloyl-*β*-D-glucose (TGG) inside the active site of M^pro^ and PL^pro^, respectively. The analysis of root mean square deviation (RMSD) **(A)**, root mean square fluctuation (RMSF) **(B)**, and radius of gyration (Rg) **(C)** for M^pro^-PGG, M^pro^-TGG, PL^pro^-PGG, and PL^pro^-TGG complexes. The molecular dynamics simulation for each system was performed for 500 ns.

Molecular dynamics simulation is pivotal for post-docking analysis, providing insights into the structural stability of the ligand-receptor complex. Here, we employed the CHARMM36m force field, which is commonly used in ligand-receptor molecular dynamics simulation study. A series of molecular dynamics simulations were performed for the SARS-CoV-2 M^pro^-PGG, SARS-CoV-2 M^pro^-TGG, SARS-CoV-2 PL^pro^-PGG, and SARS-CoV-2 PL^pro^-TGG complexes spanning a period of 500 ns. As shown in Figure 5A, the RMSD values of M^pro^ range between 1.2 and 3.0 Å in the M^pro^-PGG complex and 1.4 and 2.5 Å in the M^pro^-TGG complex, meanwhile the RMSD values of PL^pro^ range from 1.5 to 2.8 Å for the PL^pro^-PGG complex and 1.5 to 2.5 Å for the PL^pro^-TGG complex, which were witnessed to display structural stability. Moreover, we examined the RMSF for each amino acid involved in these receptor-ligand interactions. As shown in Figure 5B, it revealed that the maximum RMSF values is about 4 Å in the M^pro^-TGG, PL^pro^-PGG, and PL^pro^-TGG complexes. Nevertheless, the M^pro^-PGG complex RMSF value shows slight fluctuations. Additionally, the stability of the complex was further assessed using the Rg value, which reflects the structural compactness. The measured Rg displayed a narrow fluctuation, suggesting the good compactness of the tested compounds (Figure 5C). Generally, the simulation results indicated that the ligands, PGG and TGG, were bound to the receptor stability.

**Figure 6:**
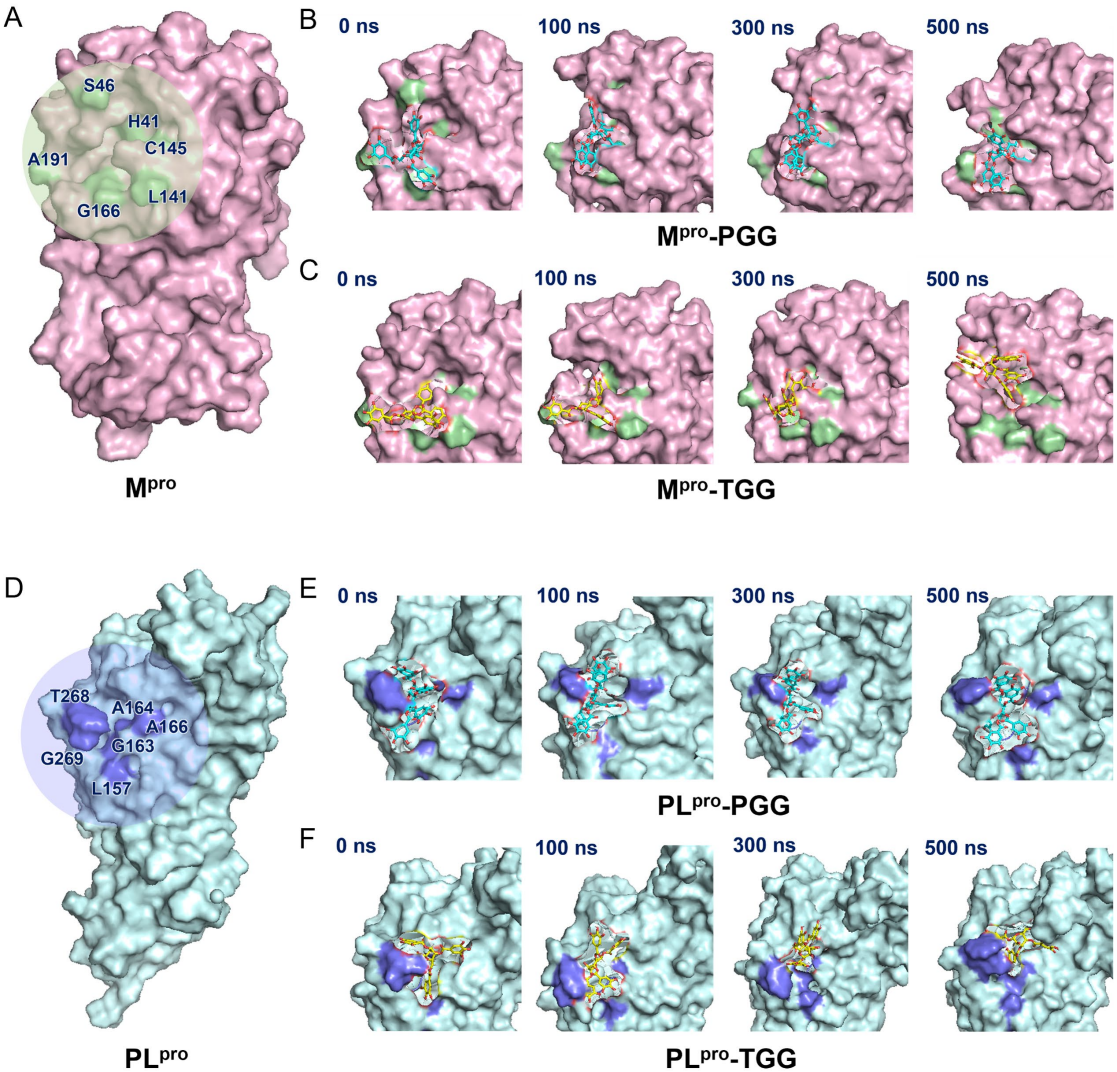
Illustration on 1,2,3,4,6-penta-*O*-galloyl-*β*-D-glucose (PGG) and 1,2,3,6-tetra-*O*-galloyl-*β*-D-glucose (TGG)‘s conformations inside the substrate-binding sites of M^pro^ and PL^pro^. **(A)** M^pro^ active site. **(B)** The surface view of M^pro^-PGG complex in molecular dynamics simulation. **(C)** The surface view of M^pro^-TGG complex in molecular dynamics simulation. **(D)** PL^pro^ active site. **(E)** The surface view of PL^pro^-PGG complex in molecular dynamics simulation. **(F)** The surface view of PL^pro^-TGG complex in molecular dynamics simulation. The snapshot was taken at 0, 100, 300, and 500 ns, respectively.

The binding sites and pocket of M^pro^ are shown in Figure 6A. Initially, PGG and TGG exhibit obvious interactions with M^pro^ and are well-encased by H41, S46, L141, C145, G166, and A191, which highlights the binding stability of the M^pro^-PGG (Figure 6B) and M^pro^-TGG complexes (Figure 6C). Similarly, the active sites and pocket of PL^pro^ are depicted in Figure 6D. Here, PGG and TGG also show evident interactions with PL^pro^ and are effectively surrounded by L157, G163, A164, A166, T268, and G269. This result demonstrates the stability of the PL^pro^-PGG (Figure 6E) and PL^pro^-TGG complexes (Figure 6F) during molecular dynamics simulation. The simulation trajectories showed that PGG and TGG could be bound stably inside the active pockets, which is consistent with the experimental results.

## 4 Discussion

With the global spread of COVID-19, it is urgent to develop specific broad-spectrum antiviral drug against SARS-CoV-2. In Chinese medicine, it gives a great opportunity to mine antiviral candidates from the structurally diverse natural products by the established method, which not only develops a strategy for rapid identification of antiviral candidate but also offers a compound library for responding to other viral outbreaks.

Among the verified therapeutic targets, M^pro^ and PL^pro^ were recognized as the most essential targets for their highly conservation in the active site from SARS-CoV-2 wide-type strain to Omicron variants (Zang et al., 2023; Rosales et al., 2024). Upon comparing M^pro^ and PL^pro^ sequences including SARS-CoV-2 wild-type, Omicron BA.5, and Omicron EG.5 with those of the currently circulating strain SARS-CoV-2 Omicron JN.1, high sequence similarity was observed (Supplementary Figures S4, S5). Given that PGG and TGG exhibit inhibitory effects on SARS-CoV-2 wild-type, Omicron BA.5, and Omicron EG.5, we hypothesize that PGG and TGG may also serve as potential inhibitors against Omicron JN.1. A number of compounds were reported to specifically inhibit M^pro^ or PL^pro^, resulting in prevention of viral replication. Importantly, the dual-target inhibitors present a promising therapeutic approach, potentially outmaneuvering drug resistance more effectively than single-target alternatives. There are a few natural compounds reported with dual-target inhibitory activity, such as schaftoside (Yi et al., 2022), chrysin 7-*O*-*β*-D-glucuronide (Yi et al., 2024), ginkgolic acid, anacardic acid (Chen et al., 2021), and (−)–epigallocatechin gallate (Wang et al., 2022) unit now. Fortunately, we found another potential dual-target compounds, PGG and TGG, as tannins. PGG possesses five galloyl substitutions, and TGG has four. PGG has been reported to have antiviral activities on herpes simplex virus type 1, influenza A virus, human respiratory syncytial virus, human rhinoviruses, and so on (Torres et al., 2017). In our study, the increased number of hydroxyl groups in the tested compounds might enhance their interaction with the protease via intermolecular hydrogen bonds. However, interactions between ligands and receptors are complex and may also arise from hydrogen bonds, *π*-π conjugation, and so on. Through systematic research, we hope to correlate the structure of tannins with their activity, which could facilitate the discovery of antiviral compounds.

Accordingly, PGG and TGG are regarded as noncovalent inhibitors. Compared with covalent inhibitors, noncovalent inhibitors often exhibit better membrane permeability and high affinity for target proteins (Zhang et al., 2015). Additionally, the reversible binding reduces the risk of undesirable toxic effects associated with interactions with host proteins and nucleic acids (El-Baba et al., 2020). PGG and TGG act as competitive inhibitors on M^pro^ and PL^pro^ of SARS-CoV-2, which are abundant in *CF*, conducing to the illumination of *CF* efficacy against SARS-CoV-2.

In order to improve the screening efficiency and reliability, we built a high-throughput screening system of virtual screening, molecular dynamics simulation combining with experimental verification, which has been demonstrated to be a successful strategy (Supplementary Figure S6). In general, PGG and TGG are considered as the promising antiviral compounds derived from *CF.* The structure of PGG and TGG also provides guidance for the design of antiviral compounds.

## 5 Conclusion

In summary, the dual-target inhibitors, PGG and TGG, were identified to act effectively against SARS-CoV-2 via targeting SARS-CoV-2 M^pro^ and PL^pro^, which satisfactorily inhibit the SARS-CoV-2 wild-type strain, Omicron BA.5 and Omicron EG.5 variant on Vero E6 cells, suggesting as the promising antiviral inhibitors. The comprehensive approach, encompassing molecular docking, FRET assay, SPR assay, antiviral activity validations, and molecular dynamics simulation, provides an effective strategy and allows us to systematically explore the antiviral bioactive compounds from *CF*, conducting to the discovery of antiviral compounds from Chinese medicine.

## Data Availability

The raw data supporting the conclusions of this article will be made available by the authors, without undue reservation.

## Footnotes

^1^ https://www.rcsb.org/

^2^ https://pubchem.ncbi.nlm.nih.gov/
