# Supplementary material for: High-throughput screening of dual-target inhibitors for SARS-CoV-2 main protease and papain-like protease from Chebulae Fructus: in silico prediction and experimental verification
Source: Front Microbiol. 2024 Dec 18;15:1510665. doi: 10.3389/fmicb.2024.1510665 (PMC11689655; doi:10.3389/fmicb.2024.1510665)
Supplement: Supplementary file 1 [file Supplementary_file_1.docx]

Supplementary Material


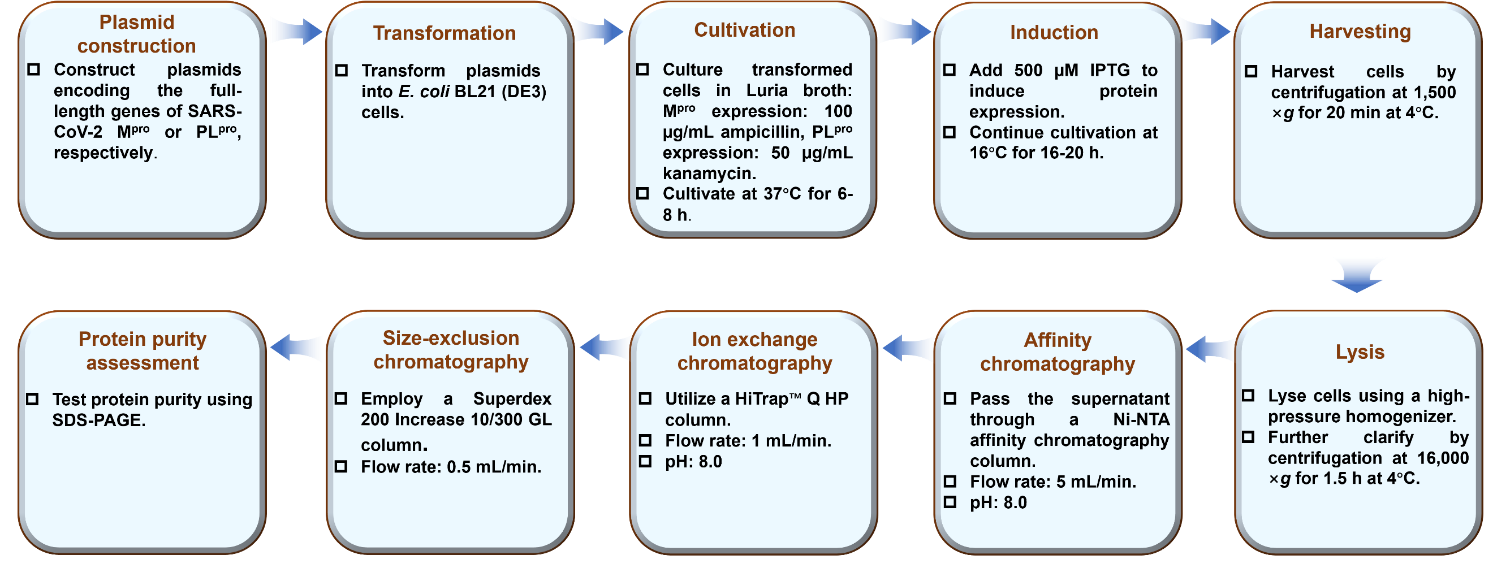


Supplementary Figure 1. The flow chart outlines the purification process for SARS-CoV-2 M^pro^ and PL^pro^.


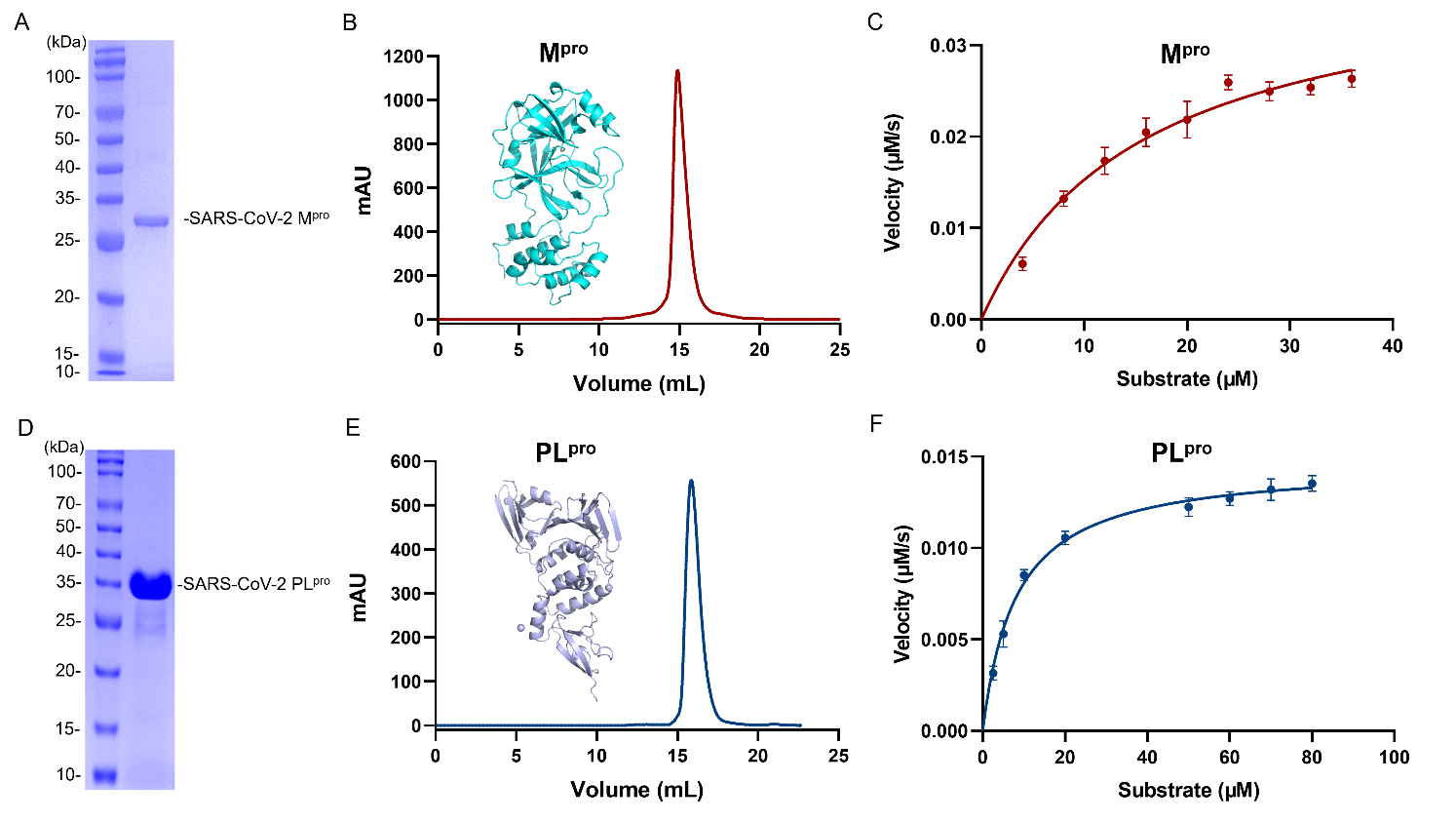


Supplementary Figure 2. The purification and evaluation of enzymatic activity for SARS-CoV-2 M^pro^ and PL^pro^. (A) The SDS-PAGE gel of SARS-CoV-2 M^pro^. (B) Size-exclusion chromatography profile of SARS-CoV-2 M^pro^. (C) Michaelis-Menten Plot of SARS-CoV-2 M^pro^. (D) The SDS-PAGE gel of SARS-CoV-2 PL^pro^. (E) Size-exclusion chromatography profile of SARS-CoV-2 PL^pro^. (F) Michaelis-Menten Plot of SARS-CoV-2 PL^pro^. (mean ± SD, *n*=3).
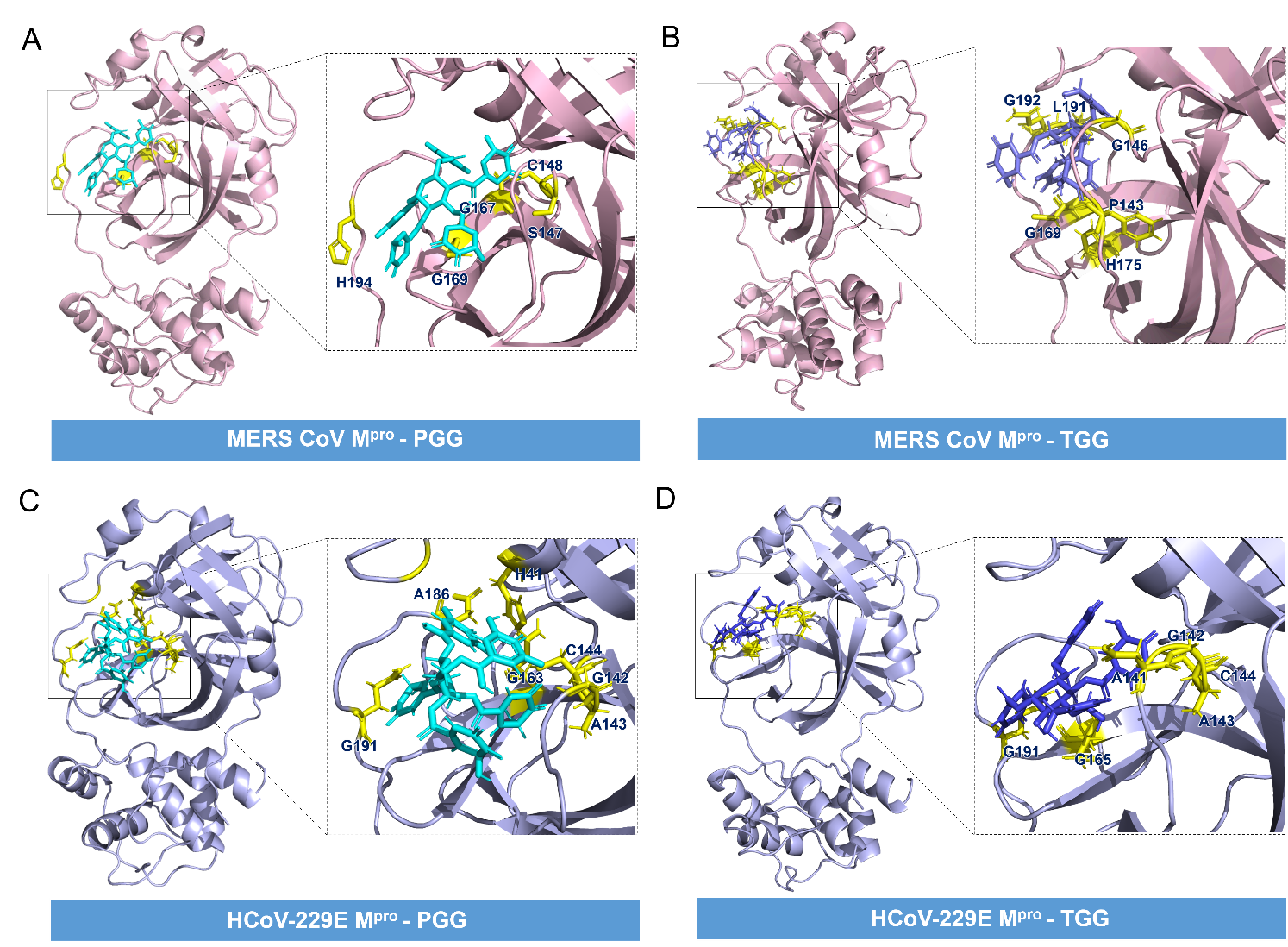


Supplementary Figure 3. Binding modes of the focused compounds and the interesting targets. (A) The predicted binding mode of MERS CoV M^pro^-1,2,3,4,6-penta-*O*-galloyl-*β*-D-glucose (PGG) complex. (B) The predicted binding mode of MERS CoV M^pro^-1,2,3,6-tetra-*O*-galloyl-*β*-D-glucose (TGG) complex. (C) The predicted binding mode of the HCoV 229E M^pro^-PGG complex. (D) The predicted binding mode of the HCoV 229E M^pro^-TGG complex.


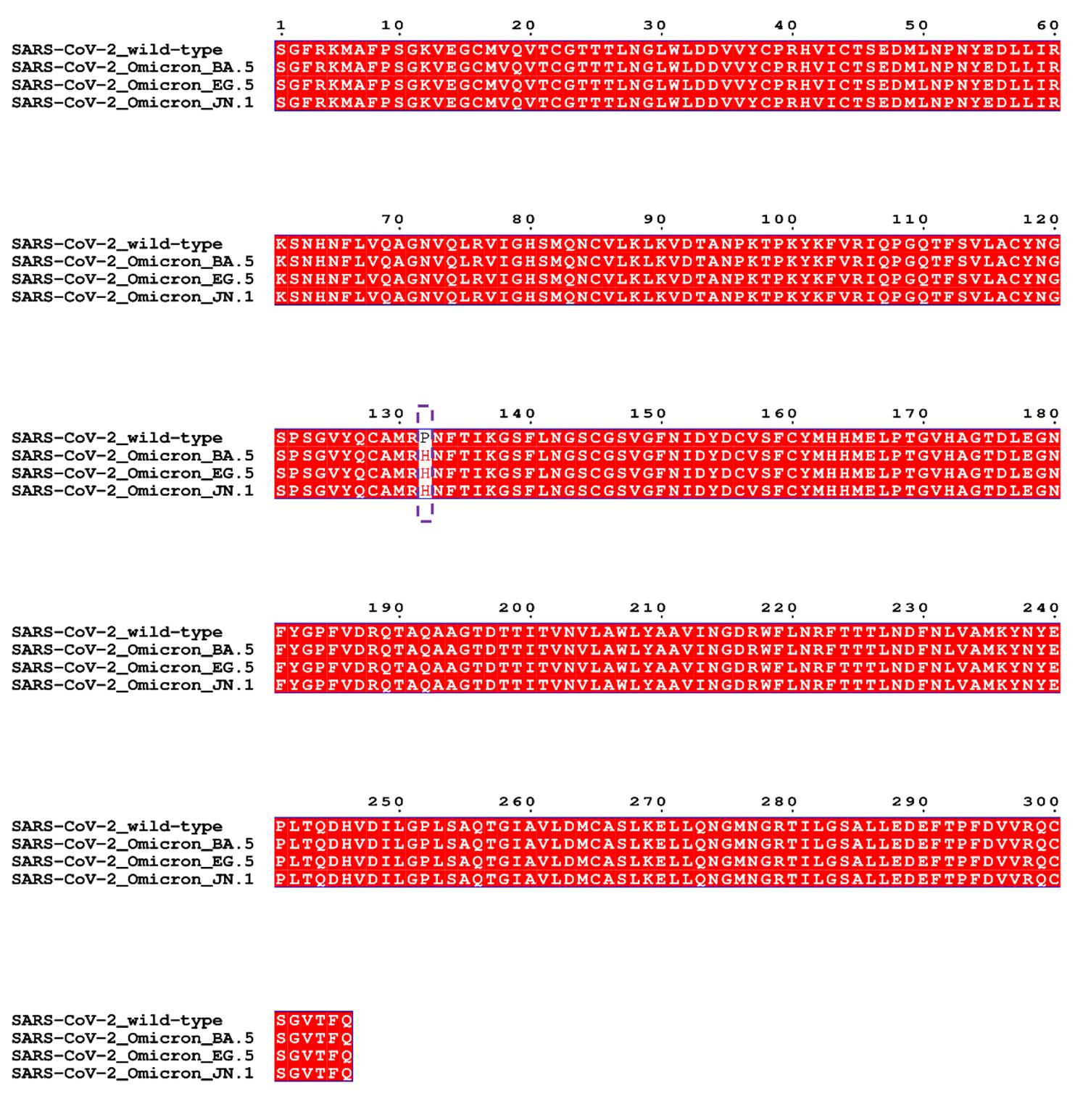


Supplementary Figure 4. Sequence alignment of M^pro^ from SARS-CoV-2 wild type, Omicron BA.5, Omicron EG.5 and Omicron JN.1.


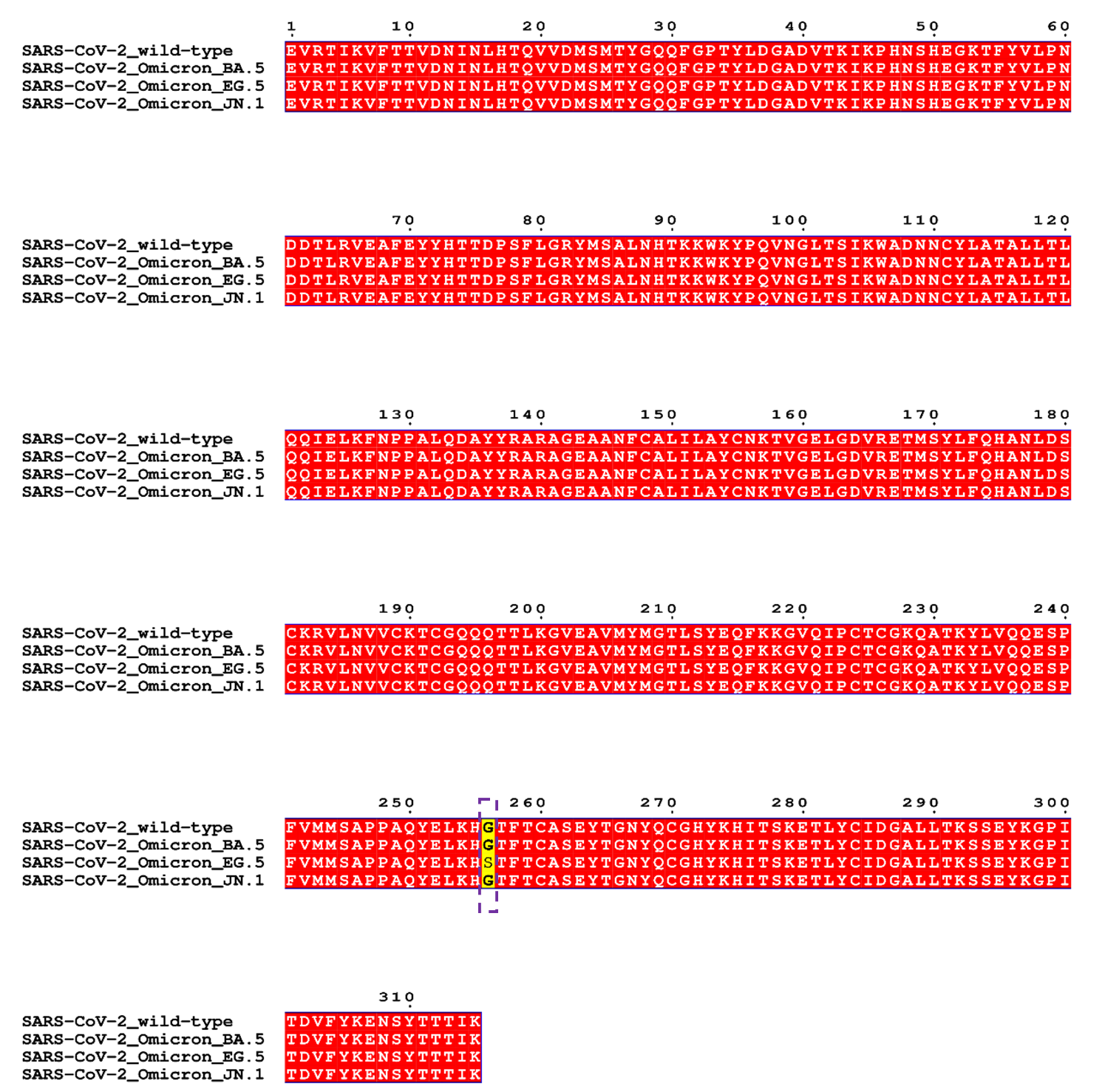


Supplementary Figure 5. Sequence alignment of PL^pro^ from SARS-CoV-2 wild type, Omicron BA.5, Omicron EG.5 and Omicron JN.1.


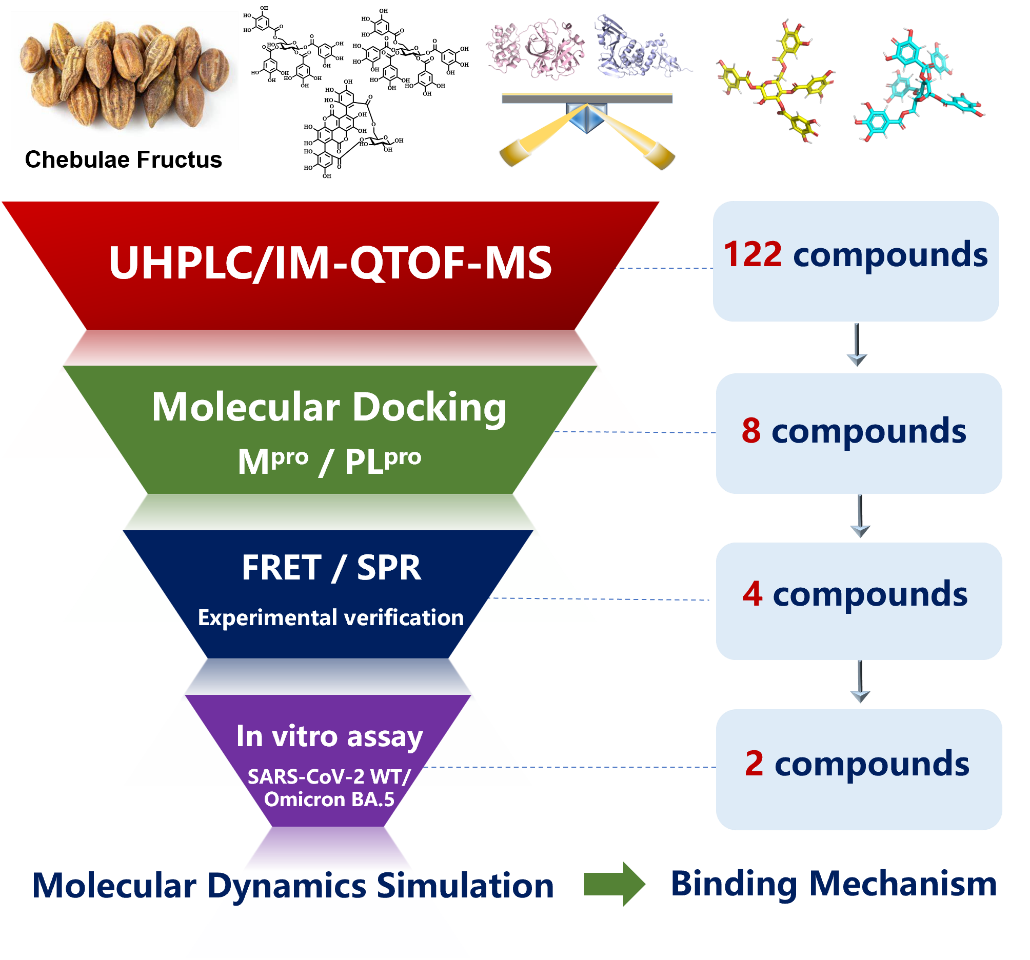


Supplementary Figure 6. *In silico* prediction and experimental verification to screen dual-target inhibitors.
